# Vertebral collapse and polymethylmethacrylate breakage after vertebroplasty: A case report

**DOI:** 10.1097/MD.0000000000016831

**Published:** 2019-08-23

**Authors:** Anquan Huang, Shenyun Fang, Liyu Wang, Renjie Xu, Jun Shen, Guoqing Zhu, Ye Miao, Tianming Zou

**Affiliations:** a Department of Spinal Surgery, The Affiliated Suzhou Hospital of Nanjing Medical University, Suzhou Municipal Hospital, Jiangsu Province; b Department of Spinal Surgery and Osteoporosis, First People's Hospital of Huzhou City, Zhejiang Province; c Department of Oncology, The Affiliated Suzhou Hospital of Nanjing Medical University, The North Area of Suzhou Municipal Hospital, Jiangsu Province, China.

**Keywords:** complications, osteoporosis, percutaneous vertebroplasty, spinal surgery

## Abstract

**Rationale:**

Vertebral augmentation has become the main treatment for osteoporotic vertebral fractures (VFs). In this article, we report a very rare case of vertebral collapse and polymethylmethacrylate (PMMA) breakage after vertebroplasty. We describe the clinical characteristics and revision surgery performed to remove the broken PMMA cement, maintain stability, and corrects the kyphotic deformity, and we analyze the possible causes.

**Patient concerns:**

A 72-year-old man who suffered back pain underwent first lumbar vertebra (L1) percutaneous vertebroplasty (PVP) due to osteoporosis and a vertebral fracture in May 2013. Postoperatively, the patient's back pain was markedly alleviated. Unfortunately, his lumbar back pain recurred in November 2015.

**Diagnoses:**

Plain radiographs showed collapse of the L1 vertebral body, breakage of the PMMA cement, and severe kyphosis at the thoracolumbar junction.

**Interventions:**

The posterior pedicle was internally fixed and an anterior artificial vertebral body implant was placed to maintain stability and correct the kyphotic deformity in a 2-step surgical procedure.

**Outcomes:**

The back pain was alleviated and the patient returned to daily life for more than two years.

**Lessons:**

This case demonstrates that PVP is not a simple minimally invasive surgery, and significant postsurgical care is necessary. The true cause of this rare phenomenon remains unclear, but the long-term use of steroids, new injuries, and poorly corrected kyphosis after PVP may play a role. Surgeons must be aware of the kinds of complications that may occur, including rare complications such as vertebral lysis.

## 1 Introduction

Due to its ability to relieve back pain effectively, vertebral augmentation, first described in 1987 by Galibert et al^[1]^ has become a widely accepted minimal method in the treatment of osteoporotic VFs and osteolytic lesions from vertebral metastases, multiple myeloma, and aggressive hemangioma. Studies by Balkarli^[2]^ and Mattie^[3]^ found that by percutaneous vertebroplasty (PVP) they were able to decrease pain more rapidly and allow patients to return to daily activities earlier than by conservative treatment for osteoporotic VFs. Although its relative safety has been proven, complications may arise.^[4]^ Saracen et al^[5]^ noted that almost 50% of patients in their large-sample study suffered complications such as intractable pulmonary embolism and cement leakage into the spinal canal, while more than 95% had no clinical symptoms.

We here report a case of an elderly man who suffered intractable back pain caused by vertebral collapse and breakage of the polymethylmethacrylate (PMMA) cement after PVP. This is a rare complication of vertebroplasty. Revision surgeries, including anterior combined with posterior surgery, were performed to restore intravertebral stability and correct the kyphotic deformity successfully.

## 2 Case description

**Figure 1:**
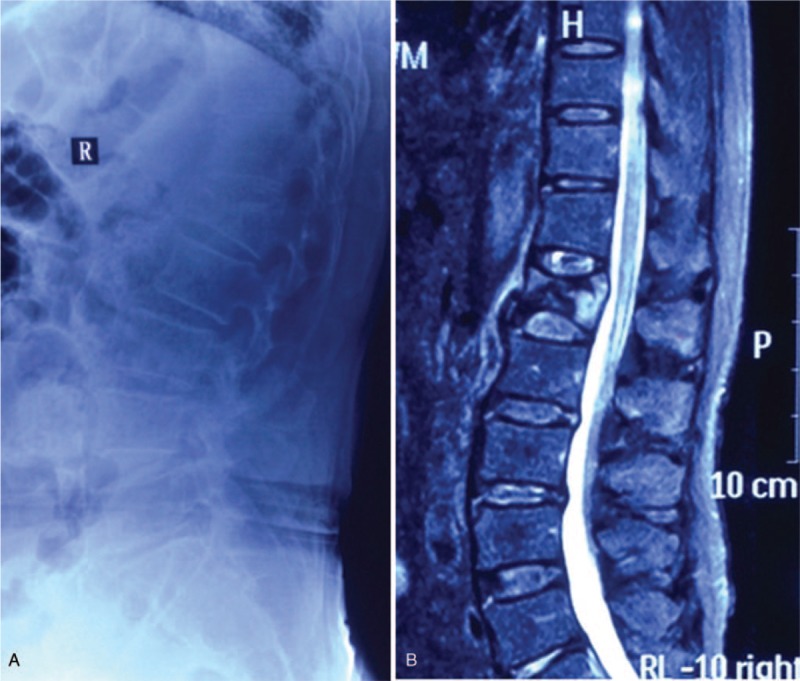
Preoperative lateral radiograph and sagittal STIR MRI (Magnetom expert 1.5 Tesla) of the lumbar spine, revealing an acute vertebral compression fracture in L1 (May 2013). MRI = magnetic resonance imaging.

**Figure 2:**
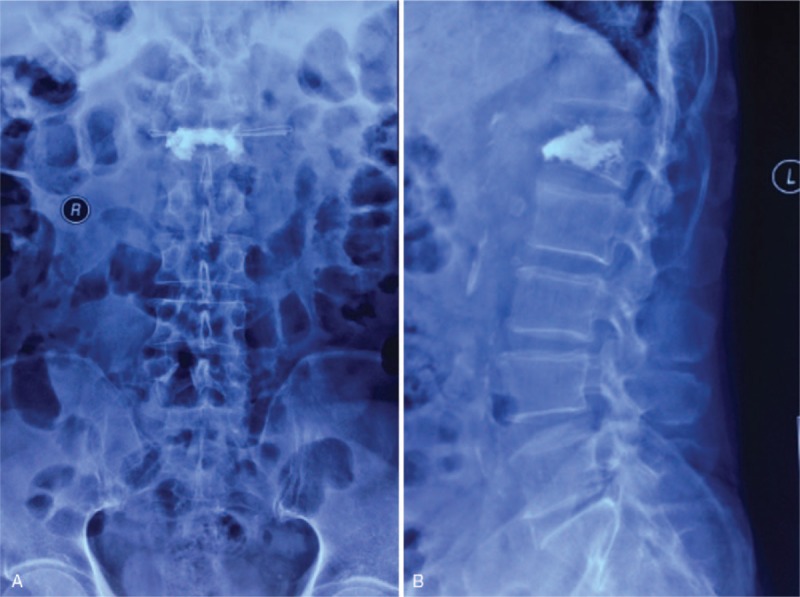
Postoperative anteroposterior and lateral radiograph of L1, demonstrating that a total of 4.0 mL PMMA was bilaterally injected through the vertebral pedicle, but the kyphosis had not been corrected (May 2013). PMMA = polymethylmethacrylate.

In May 2013, a 72-year-old man suffered back pain after a slight injury caused by a fall at home. X-ray imaging revealed a compression fracture of the first lumbar vertebra (L1) vertebra, and magnetic resonance imaging (MRI) revealed an acute fracture in the L1 vertebra (Fig. 1). He underwent PVP, and a total of 4 mL polymethyl was bilaterally injected into the vertebra through the vertebral pedicle, under local anesthesia (PMMA [Heraeus Medical GmbH, Osteopal V; Heraeus Medical GmbH, Wehrheim, IN, Germany]). Postoperatively, the patient's backache had been alleviated considerably: the visual analog scale (VAS) score improved from 7 to 2, and the Oswestry disability index (ODI) score had decreased from 75 to 15 after the first day (Fig. 2).

**Figure 3:**
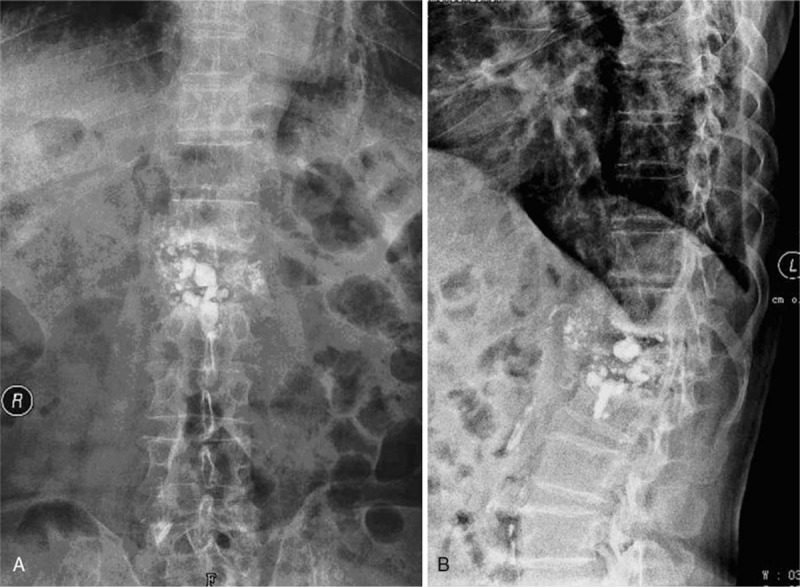
Plain radiograph showing L1 vertebral collapse and PMMA breakage, with a kyphosis angle of 25° (March 2016). PMMA = polymethylmethacrylate.

In November 2015, the patient's lumbar back pain recurred. The pain was endurable at first, so he rested at home. The back pain gradually worsened, however, especially when he was standing or sitting. The patient sought treatment at our hospital, and further plain radiographs showed that the L1 vertebra had collapsed and the PMMA cement was comminuted (Fig. 3). He was then admitted to the hospital for further treatment.

The patient had a 10-year history of rheumatoid arthritis (RA) and he received 1 dose of oral methylprednisolone daily (10 mg). He had also undergone a gastric resection for early-stage gastric cancer 6 years earlier. He showed good postoperative recovery and pathology showed no metastasis.

Physical examination revealed marked tenderness on palpation of the posterior spinous process of L1. Pain did not radiate to the lower limbs. The strength and feel of the lower limbs were normal. The joints of the hands and feet showed visible deformity.

The patient's temperature was normal. Further evaluation of the C-reactive protein level, erythrocyte sedimentation rate, white blood cell count, and T-SPOT test preliminarily ruled out vertebral osreomyelitis, abscess formation, and tuberculosis. Tumors such as myeloma and metastatic tumors were also considered. ALP, AFP, CEA, and CA-199 indices were normal too. The rheumatoid factor was 1120 IU/mL.

**Figure 4:**
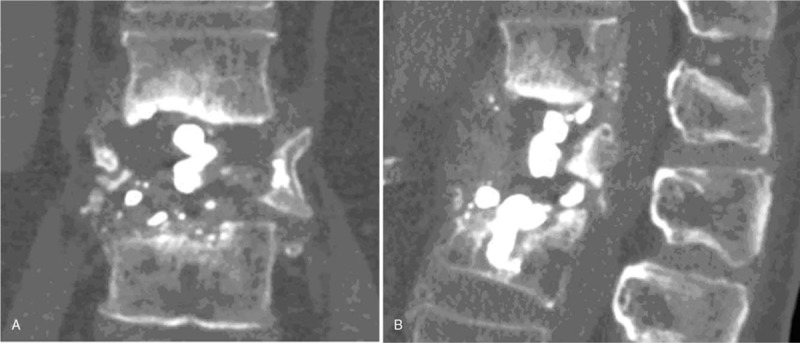
Computed tomography reconstruction of the L1 vertebra showing vertebral collapse of L1 after vertebroplasty, with broken PMMA pieces embedded in the L2 vertebra. PMMA = polymethylmethacrylate.

**Figure 5:**
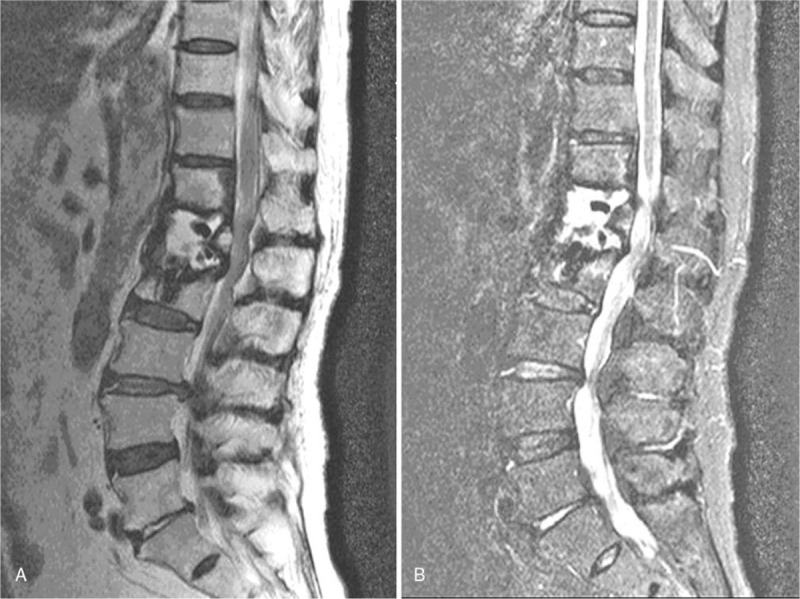
Sagittal T1-weighted and STIR MR images (Magnetom expert 1.5 Tesla) showing low and high signal intensity on L1, respectively.

X-ray images showed the Cobb angle was 35° and 20° in flexion and extension positions, respectively. Further computed tomography examination revealed osteolysis of the L1 vertebra, and pieces of PMMA cement were embedded in the L2 vertebral body (Fig. 4). Sagittal T1-weighted and STIR MR images showed low and high signal intensity for L1, respectively, which indicated L1 was full of liquid (Fig. 5). His bone mineral density, measured at the proximal femur and L2–4, was 0.653 g/cm^2^ (T score: − 2.5, Z score: − 1.1) and 1.515 g/cm^2^ (T score: 3.6, Z score: 4.4), respectively.

**Figure 6:**
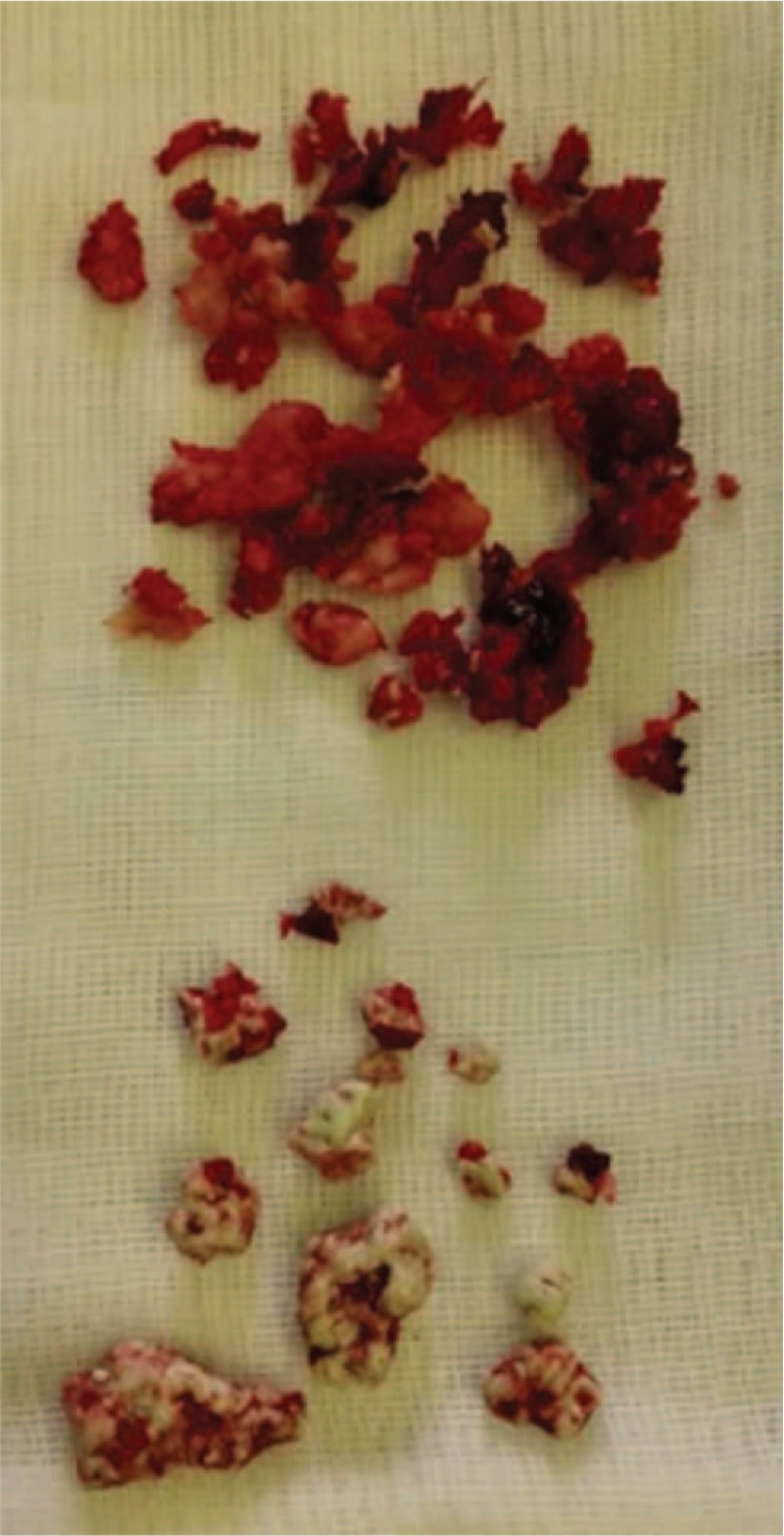
Broken bone and PMMA were removed during surgery. PMMA = polymethylmethacrylate.

**Figure 7:**
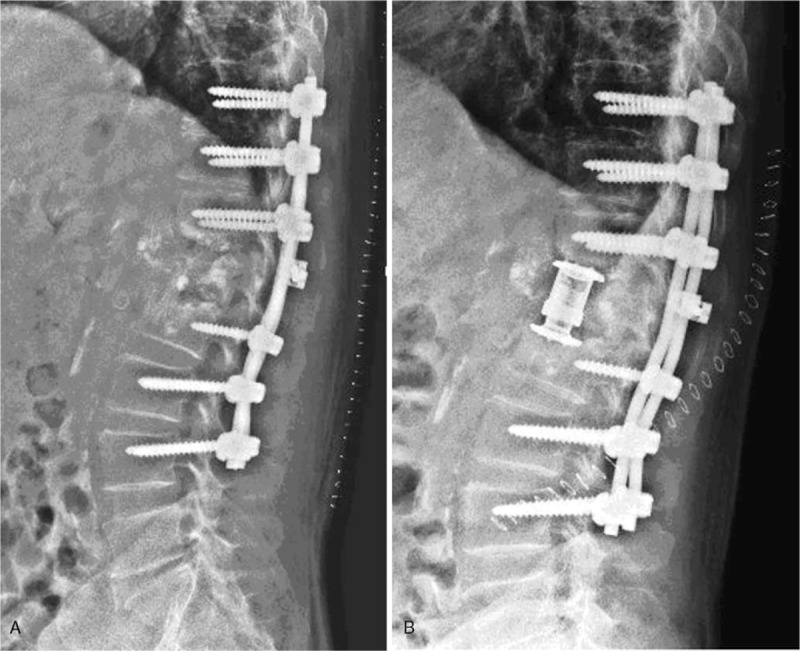
Lateral X-rays showing 2 steps of posterior pedicle internal fixation and anterior artificial vertebral body implantation immediately after reconstruction.

The patient underwent posterior pedicle internal fixation surgery in a single-stage operation, and most of the broken PMMA was removed through a gap on the left side of the L1 transverse process and the lateral pedicle (Fig. 6). A light-yellow clear liquid was observed in the L1 vertebra, and the liquid was sent for microbial analysis. Then the vertebra was repeatedly irrigated using a hydrogen peroxide solution and diluted iodine. The antibiotic cefathiamidine was used to prevent postoperative infection. The microbial culture was negative, and pathological examination showed broken bone tissue and large amounts of cellulose fiber. The 2-stage anterior side of the artificial vertebral body was implanted 2 weeks later (Fig. 7). The patient has been followed up for more than 2 years. He has recovered well, the pain is relieved, and the ODI score decreased from 98 to 27.

## 3 Ethics statement

Our hospital does not require formal ethical approval for case reports with written consent. Written informed consent was obtained from the patient and his wife for publication.

## 4 Discussion

In this report, we present a rare case of symptomatic vertebral collapse and PMMA breakage after PVP. Back pain and limitation of daily activities were addressed by further focal debridement, transvertebral screw fixation, and an artificial vertebral implant. The patient returned to normal daily life after treatment.

Osteoporosis is a normal physiological phenomenon associated with aging. Some diseases, such as RA, nephrotic syndrome (NS), systemic lupus, and erythematosus (SLE), can accelerate bone loss. Vertebral osteoporotic fractures are among the most common osteoporotic fractures, and vertebroplasty is considered by numerous surgeons to be a safe and effective procedure for treating this kind of symptomatic fracture. Despite its high success rate, numerous complications can occur in up to 70% of cases. However, most complications have no clinical symptoms, according to many medical studies.^[5–6]^

Complications can be divided into immediate complications, such as extraossal cement leakage and cement embolism, and delayed complications, such as vertebral collapse and infection. Immediate complications can be prevented by the use of a hydraulic pump, precise placement of the punctuation needle, and intraoperative fluoroscopy.^[7]^ Rapan et al^[8]^ reported that the use of high-viscosity cement can minimize the risk of extraossal cement leakage. Kyphoplasty has a lower risk of cement leakage than vertebroplasty, and it can reduce the risk of pulmonary embolism and nerve compression.^[9]^ Nevertheless, 1 study found that PVP performed after osteoporotic VFs cannot significantly improve kyphosis.^[2]^ Infection after vertebroplasty rarely occurs. Liao et al^[10]^ reported the incidence of infection after vertebral augmentation is between 0% and 1%. Infection was ruled out in the present case by laboratory and pathological examination.

The relationship between vertebroplasty and subsequent fractures remains unconfirmed. Some authors^[11]^ confirmed that new VFs are a natural result of osteoporosis and have no significant relationship with fractures after vertebroplasty. Cao et al^[12]^ came to the same conclusion. However, they pointed out that 3 primary factors, that is, low bone mineral density, steroid usage, and the presence of multiple treated vertebrae, may be associated with a higher incidence of new fractures after vertebroplasty. Some other important factors, such as kyphosis correction and intradiscal cement leakage, were not found to be related to new fractures. Ahrar declared that the adjacent fracture risk is related to the filling rate of the treated vertebrae, which may influence the force exerted on the adjacent level due to the cement burden.^[13]^ Nevertheless, the triggering role of PVP in new osteoporotic fractures is still a matter of debate. Considering the biomechanics of the spine, Nagaraja et al^[14]^ hypothesized vertebroplasty of the vertebrae may increase compression on adjacent vertebral bodies and intervertebral discs, especially in severely osteoporotic women.

The precise cause of vertebral collapse remains an enigma, and there are several underlying factors that may be related to this particular case of vertebral collapse and PMMA breakage. First, due to the long-term use of glucocorticosteroids by this patient, it was difficult to treat osteoporosis or even prevent progression. Second, back pain and disability were reduced by vertebroplasty but without correcting kyphosis. Therefore, the disturbed biomechanics was not recovered, and the L1 vertebra was loaded with more weight. Finally, new fractures of L1 may have occurred during the 18 months after vertebroplasty, when the patient no longer received intervention treatment, such as immobilization or surgery. Sun et al^[15]^ reported 3 new fracture signs on the other side of the previously augmented bodies in their case report.

To treat the rare complication, a 2-step operation was performed. Yoshii et al^[16]^ recently published a case of cemented and adjacent vertebral collapse (L3 and L4) 6 months after PVP with calcium phosphate cement. He also employed the salvage procedure of anterior-posterior combined reconstruction to correct the severe kyphotic deformity and effectively maintain balance after the failed PVP.

According to the research reported by Jeon et al, the height of a vertebra can improve by an average of 21% after kyphoplasty.^[17]^ For patients with a long history of hormone use, who have concomitant diseases, such as RA, NS, and SLE, and who suffer from osteoporotic VFs, kyphoplasty may be a better choice due to the better restoration of the vertebral height and local kyphotic angle corrections.^[18]^ It is important to inform patients who undergo PVP for osteoporotic VFs of the possible complications, which include re-fracture, before they undergo the surgery. It is also necessary to advise the patients to receive regular anti-osteoporosis treatment after surgery.

## Author contributions

**Conceptualization:** Tianming Zou.

**Investigation:** Anquan Huang, Renjie Xu, Jun Shen, Guoqing Zhu, Ye Miao.

**Supervision:** Tianming Zou.

**Writing – original draft:** Anquan Huang.

**Writing – review & editing:** Shenyun Fang, Liyu Wang, Renjie Xu, Jun Shen, Guoqing Zhu, Ye Miao, Tianming Zou.
